# Peripheral Helper T Cell Responses in Human Diseases

**DOI:** 10.3389/fimmu.2022.946786

**Published:** 2022-07-08

**Authors:** Hiroyuki Yoshitomi

**Affiliations:** Department of Immunology, Graduate School of Medicine, Kyoto University, Kyoto, Japan

**Keywords:** peripheral helper T cell (Tph), peripheral immune response, autoimmune disease, somatic hypermutation, B cell

## Abstract

A series of rheumatoid arthritis (RA) studies established a PD-1^hi^CXCR5^-^CD4^+^ T-cell subset that was coined peripheral helper T (Tph) cells. CXCL13 production is a key feature of Tph cells and may contribute to the formation of tertiary lymphoid structures (TLS) in inflamed tissues. In addition, Tph cells provide help to B cells *in situ* as efficiently as follicular helper T (Tfh) cells, and these features would implicate Tph cells in the pathogenesis of RA. Subsequent studies have revealed that Tph cells are involved in various human diseases such as autoimmune diseases, infectious diseases, and cancers. Although the analysis of human immunity has various limitations, accumulating evidence demonstrated the expansion of B cells with low somatic hypermutation and a link between TLS and immune functions in these diseases. We discuss about the emerging roles of the Tph cell and its relevant immune responses in peripheral tissues including B-cell expansion with atypical features.

## Introduction

The role of CD4^+^ T cells in the pathogenesis of autoimmune diseases has attracted attention based on their association with autoantibodies and MHC class II. A series of studies on inflamed joints of rheumatoid arthritis (RA) established a population of PD-1^hi^CXCR5^-^CD4^+^ T cells that was coined peripheral helper T (Tph) cells (1–3). Tph cells intensively secrete CXCL13, a chemokine crucial for the formation of lymphoid follicles at inflammatory sites and contribute to the establishment of acquired immunity by providing help to B cells *in situ*. Tph cells have been reported to be involved in the pathogenesis of not only RA but also various autoimmune diseases, infections, and malignant tumors in humans. Furthermore, it is becoming clear that in addition to Tph cells, B cells with atypical features are involved in immune responses in the peripheral tissues of these diseases. This review describes the emerging roles of the Tph cell and its relevant immune response including B-cell expansion with atypical features in various human diseases.

### Tph Cells in RA

In the 2000s, a diverse study showed that Th17 cells, which produces inflammatory IL-17 family cytokines, play crucial roles in mouse models of autoimmune diseases (4). This suggested the importance of the inflammatory aspect of CD4^+^ T cells in autoimmune diseases. In human RA, however, Th17 cells are not increased in the blood and joints (5), and IL-17-neutralizing therapy was not sufficiently effective unlike in psoriatic arthritis (6). Therefore, the pathogenic function of CD4^+^ T cells needs to be viewed from a different aspect than IL-17 production.

RA is characterized by the production of autoantibodies such as rheumatoid factor (RF) and anti-citrullinated antibodies (ACPA) (7) and by hyperplasia of synovial tissue accompanied by a SLO-like structure, tertiary lymphoid structure (TLS) (8). Therefore, pathogenic CD4^+^ T cells of RA were supposed to support these features. Exploring T-cell subsets in RA-inflamed joints identified a CD4^+^ T-cell population that intensively produces CXCL13 (1) at levels sufficient to recruit B cells (2). scRNA-seq of RA synovial tissue showed that CD4^+^ T cells were the main source of CXCL13 (9). These, together with the data that ectopic CXCL13 expression is sufficient for TLS formation (10), suggest that RA synovial CXCL13-producing CD4^+^ T cells are involved in TLS formation. Given that in SLO, FDC, a stroma cell, forms germinal center (GC) through CXCL13, it is surprising that T lymphocytes have similar properties in inflamed tissues.

Another study revealed that these CXCL13-producing CD4^+^ T cells have further immune functions associated with inflammatory tissues. Tph cells localize to inflammatory tissues and provide help to B cells *in situ* (3). The expression of chemokine receptors is a key difference between Tph and Tfh cells; Tph cells do not express CXCR5, crucial for the migration into GCs, but express inflammation-related chemokine receptors including CCR2 and CCR5 and infiltrate into inflammatory tissues (3). At the same time, Tph cells partly show features of Tfh cells such as ICOS, CD40L, and IL-21 and provide help to B cells. Such cells that can provide help to B cells are supposed to have experienced antigens. Consistently, most of CXCL13-producing CD4^+^ T cells are memory T cells that lack CD45RA expression and express activation markers such as CD69 and particularly PD-1. Thus, CXCL13-producing cells and PD-1^hi^CXCR5^-^ Tph cells were largely overlapping in RA-inflamed joints.

### CXCL13 Production and Regulation by Tph Cells

TLSs at inflammatory sites may be partly attributed to CXCL13 produced by Tph cells. The frequency of TLS in the synovium of seropositive-RA is 10%–40%, with other cases involving only loosely organized lymphoid aggregates without TLS (8). Loose lymphoid aggregates and TLS are not necessarily mutually exclusive in the RA synovium (8) or malignant tumors; rather, they might be a continuous spectrum of TLS states (11). Therefore, Tph cells may contribute to the formation of these less organized aggregates as well as to TLS formation. When inflammation occurs in normal tissues, locally activated Tph cells intensively produce CXCL13, which may contribute to TLS formation in peripheral tissues. Subsequently, TLS supports further immune responses *in situ*. Therefore, CXCL13 regulation in T cells at inflammatory sites is a key to the peripheral immune responses. The most crucial factor for CXCL13 induction is TGF-β, which activates SMAD2/3 and upregulates transcription factor Sox4 in CD4^+^ T cells (12, 13). Proinflammatory cytokines such as TNF and IL-6 are associated with sustained CXCL13 expression: proinflammatory cytokines support the long-term (1-week) production of CXCL13 from CXCL13-producing CD4^+^ T cells in a memory state reactivated by TCR stimulation (2). On the other hand, the IL-2/STAT5 pathway and expression of Blimp1 suppress CXCL13 expression (12, 13). Presumably, this inhibitory mechanism may exist to stop new TLS formation to prevent excessive immune response once the *in situ* immune state is activated enough to produce IL-2. A similar negative regulation is also observed in Tph cells in several ways. PD-1 is a main surface marker of Tph and Tfh cells as well as a marker of exhausted cells and provides inhibitory signal to the cells. Although Tph and Tfh cells are not supposed to be exhausted because of their sufficient B-cell-helper activities (3), PD-1 signaling still provides inhibitory signals to Tph cells; blocking PD-1 signaling increases cell proliferation and CXCL13 production in Tph cells (14). The activation of Tph and Tfh cells by the blockade of PD-1 signaling may also be involved in immune-related adverse events (irAE) of cancer patients receiving PD-1-targeted cancer immunotherapy. Tfh or Tph cells might be associated with the development of autoantibodies including anti-thyroglobulin antibody which is significantly related to the persistent thyroid dysfunction of patients with irAE (15). Although the physiological or pathogenic roles of PD-1 in Tph cells remain to be determined, PD-1 might play roles as a kind of safeguard against the excessive function of Tph cells. Thus, the production of CXCL13 is one of the initial key functions of Tph cells and is regulated by various pathways.

### Localization of Tph Cells in Inflamed Sites

Tph cells produce CXCL13 but, unlike Tfh cells, do not express its receptor CXCR5. Therefore, Tph is supposed to localize to the periphery of the TLS rather than to its center. Instead of CXCR5 expression, Tph cells express chemokine receptors such as CCR2 and CCR5 in RA (3) and CXCR3 (16, 17), CX3CR1 (18), and CCR9 (19, 20) in other diseases including SLE, IgG4-related disease, and Sjögren’s syndrome. Furthermore, some Tph cells express CCR7 (1), a marker of central memory T cells that can migrate into SLOs. Consistently, RA synovial tissue expresses CCL19 and CCL21, the ligands for CCR7 (21). These indicate that synovial tissue acquired SLO-like properties that allow migration of CCR7-positive cells such as naïve T and B cells, central memory T cells, mature dendritic cells, and plasmacytoid dendritic cells. Thus, Tph cells localize in inflamed tissues according to their respective chemokine gradients.

### Tph Differentiation

Since CXCL13 is a key feature of Tph cells, TCR stimulation and TGF signaling also play key roles in Tph differentiation (12, 13). The importance of this signaling is also shared between Tph and Tfh cells. The inhibition of differentiation by IL-2 signaling is also shared in Tph and Tfh cells (22–24). IL-12/23 signaling may be involved in Tfh differentiation; IL-12/23 signaling promotes the expression of CXCR5 and BCL6 (22, 23). Although the role of this signaling in Tph-cell differentiation is still unclear, the insufficient clinical effect of IL-12/23 inhibitors in RA (6) implies a lesser role for IL-12/23 in Tph-cell differentiation. It is interesting to note where the differentiation of Tph cells takes place. In the very early stages of diseases, before the formation of TLS in peripheral tissues, immature Tph cells may develop accompanied by Tfh-cell differentiation in SLOs. The presence of Tph-like cells that substantially share TCR with Tfh cells in lymph nodes of patients with HIV supports this development (25). Such immature Tph cells are considered to migrate to peripheral inflammatory tissues and to contribute to TLS formation *in situ*. After the establishment of TLS in inflamed sites, relatively naïve-state T cells including CCR7-positive CD4^+^ T cells may migrate into TLS and locally differentiate into Tph cells.

### B-Cell Help of Tph Cells

Tph cells express Tfh-cell signatures related to B-cell-helper function such as IL21, ICOS, Maf, and SLAM family (3, 24). Despite not expressing BCL6, Tph cells provide help to memory B cells as efficiently as Tfh cells (3). Transcription factor Maf is involved in the expression of genes such as *SH2D1A*, *IL21*, *ICOS*, and *SLAMF6* (13, 26) and in B-cell-helper function (27). On the other hand, Tph cells are supposed to provide lesser help to naïve B cells than Tfh cells; naive B cells cocultured with Tph cells do not undergo class switching, whereas Tfh can induce class switching in naïve B cells (28). Although the molecular mechanism for this difference is not clear, Tph cells would preferentially provide help to memory B cells already sensitized.

### Features in Target B Cells of Tph Cells

Tfh cells migrate to the B-cell zone and exert a GC response to GC-B cells. Both Tfh and B cells co-localize according to the chemokine gradient of CXCL13 with the guide of CXCR5 and interact with each other. Similarly, Tph cells would target B cells that coexist in peripheral tissues. “CXCR5 negative” B cells are more likely to coexist and interact with Tph cells. Many of the B cells that PD-1^hi^ T cells (primarily Tph cells) contact with outside of lymphoid aggregates in the RA synovium have been shown to be CXCR5 negative (3). This suggests that CXCR5^-^ B cells in the CXCL13 gradient localize outside, rather than inside, the lymphocyte aggregates and, consequently, can co-localize with Tph cells present outside the lymphoid aggregates. DN2 cells are CXCR5-negative human B cells defined by Jenks et al. as IgD^-^CD27^-^CXCR5^-^ cells (29). DN2 cells harbor the properties of CD21^-^CD11c^+^Tbet^+^, overlapping with age-associated B cells (ABC) (29–31).

Somatic hypermutation (SHM) of BCR is an important feature for antibody affinity maturation (32). It is not clear, however, whether SHM occurs in the same way as GCs in human B cells differentiating at extrafollicle or peripheral tissues. In mouse spleen, GC and extrafollicle B cells have been similarly somatically hyper-mutated (33). On the other hand, various studies have shown that B cells in patients with inflammatory diseases have less SHM. DN2 cells in SLE have less SMH than other types of B cells (29). Similarly, SHM of IgG sequences from B cells of colon tissues is lower in patients with ulcerative colitis (UC) than in healthy individuals (34). These collectively indicate that human B cells differentiated at extrafollicle or peripheral tissues have less SHM than GC-B cells. The clonal expansion suggests that these B cells develop *in situ* with the help of T cells, most likely with that of Tph cells. The factors regulating Tph targeting might depend on disease conditions. In UC, CD27^+^ switched memory B cells expand with lower SHM in the inflamed colon rather than DN2 cells (34).

**Figure 1:**
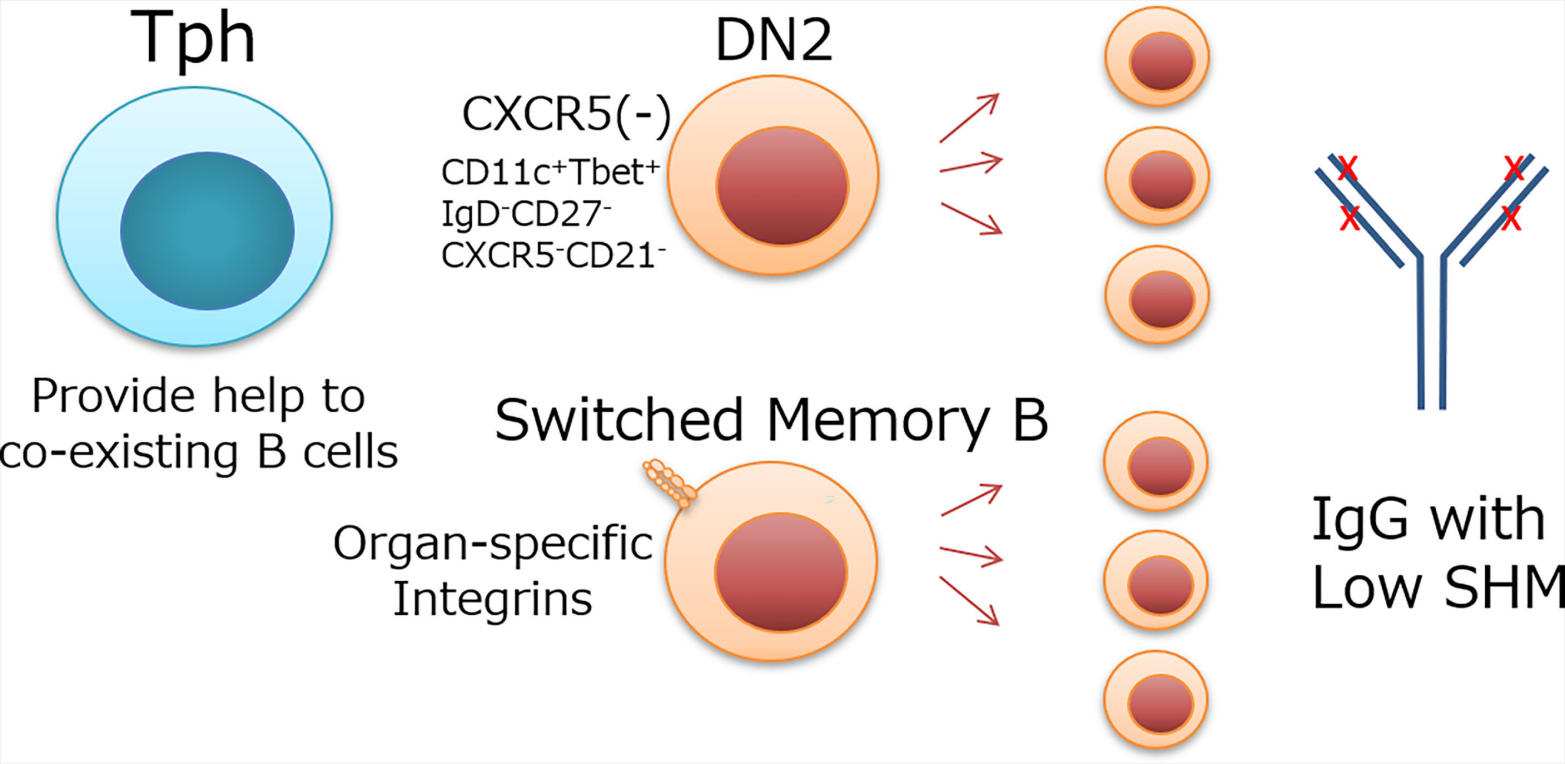
Possible target B cells of Tph cells and their features. Tph cells localize in peripheral tissues or extrafollicle and provide help to coexisting B cells. DN2 cells, which do not express CXCR5 like Tph cells, and switched memory B cells, which express organ-specific integrins and coexist with Tph cells in inflammatory tissues, are candidate target cells for Tph cells. These B cells expand with low SHM Ig.

The mechanism and significance of B-cell differentiation with low SHM is unknown, but there would be three possibilities. 1) The BCRs have sufficient affinity for self-antigens despite low SHM, and further affinity maturation is not required. 2) Ubiquitous autoantigens allow the proliferation of B cells harboring BCR with relatively low affinity and SHM. 3) Tph cell-dependent B-cells help preferentially support B-cell development without SHM unlike Tfh cells. Although the immunological significance of B-cell expansion with low SHM remains to be determined, B cells targeted by Tph cells appear to exhibit low SHM (**Figure 1**). Such expansion may be related to a rapid but less specific immune response in peripheral tissues of human diseases.

### Tph Cells in Mouse Models

Studies on the role of Tph cells in mouse models are limited unlike human Tph-cell studies. Mouse PD-1^hi^CXCR5^-^ Tph cells produce IL-21 as well as human Tph cells and have been shown to be involved in vascular endothelial inflammation (35). On the other hand, the mouse Tph model has been considered challenging because mouse CD4^+^ T cells have not been supposed to produce CXCL13. Probably due to advances in analytical techniques, however, the expression of CXCL13 has been reported in CD4^+^ T cells present during aging and in tumor TLSs. In aged kidney, CXCR5^-^PD-1^+^CD153^+^ cells expressed significantly more *Cxcl13* mRNA than PD-1-negative cells (36). In the tumor, deficiency of Satb1 intensively enhanced *CXCL13* expression by mouse Tfh cells within intratumor TLS (37). It is still unclear whether mouse T cells are the main source of CXCL13 in inflammatory tissues, as observed in scRNA-seq of the human RA synovium. To investigate the *in vivo* role of Tph cells or TLS, mouse Tph models that reflect human Tph cells should be developed.

### Tph Responses in Autoimmune Diseases

Tph cells have been reported to be associated with various autoimmune diseases as well as RA. Although functions of Tph cells in peripheral tissues were investigated in a limited number of autoimmune diseases such as UC and juvenile idiopathic arthritis (34, 38), circulating Tph (cTPh) cells with the definition of PD-1^hi^CXCR5^-^ or PD-1^+^CXCR5^-^ in peripheral blood may reflect the systemic status of Tph cells. This concept would be analogous to the analysis of circulating Tfh (cTfh) cells that had been established in a variety of human diseases (39). cTph cells retain some degree of tissue Tph-cell phenotype such as *MAF*, *IL-21*, *CXCL13*, and *TIGIT* (3, 27, 28). Inflammatory environment-dependent *CXCL13* and *IL-21* are reduced in cTph cells compared to tissue Tph cells, while cTPh cells provide help to memory B cells as efficiently as tissue Tph cells (3, 28). cTph cells are increased in autoantibody-positive RA patients, whereas they are not increased in seronegative RA and spondyloarthritis patients (3). Furthermore, their increase has been reported in other autoimmune diseases, such as SLE (16, 17, 27), IgG4-related diseases (18, 40), type I diabetes (41), systemic sclerosis (42), primary biliary cirrhosis (43), Sjogren’s syndrome (44, 45), and IgA nephropathy (46). Correlations between cTph-cell frequency and disease activity, such as SLEDAI in SLE (16, 17, 27), and worsening renal function in IgA nephropathy (46) also support the relationship between Tph cells and autoimmune disease pathogenesis. Similarly, autoantibodies such as anti-dsDNA antibodies in SLE (17, 27) and IAA, IA-2A, and GAD autoantibodies in type I diabetes (41) correlate with the frequency of cTph cells. However, a strong correlation in frequencies of cTph and cTfh (47) makes it difficult to infer which cell is directly involved in the differentiation of B cells in autoimmune diseases. As mentioned in a previous part, SHM analysis of Ig would provide speculation on this. SHM is lower in the overall IgG of patients with RA than in that of healthy controls (48). This could be interpreted as Tph cells providing more help to B cells than Tfh cells in RA. Similar observations are reported in the inflamed colon of UC; switched memory B cells oligoclonally expand in the inflamed colon with low SHM (34). Colocalization of B cells and CXCL13-producing CD3^+^ T cells shown by multiple immunofluorescent stainings suggest that Tph cells provide help to switched memory B cells in UC-inflamed colitis (34). Interestingly, integrin β7-positive gut-homing B cells are also upregulated with lower SHM in the peripheral blood of UC patients than in that of healthy controls (34), implying the involvement of molecules that regulate organ-specific homing in Tph targeting. In patients with SLE, DN2 cells have been shown to expand with low SHM (49). Indeed, blood B cells with a high expression of CD11c, a signature of DN2 cells, correlate well with the frequencies of cTph cells in SLE (27). Considering the lack of obvious proliferation of DN2 in inflamed UC colitis (34), factors that regulate the coexistence with Tph cells depend on human diseases or conditions (**Figure 1**). Interestingly, these studies showed that SHM decreases more in IgG than in IgA at these conditions. There might be a tendency for class switch to IgG in B-cell differentiation without SHM in these diseases (34, 48). Frequencies of cTph in IgA nephropathy are correlated with disease progression (46). Whether this association is an apparent association due to the strong correlation of Tph and Tfh frequencies or due to the coexistence of Tph cells and IgA-producing B cells awaits further analysis. On the other hand, it has been reported that ACPA, an antibody specific for RA, has high SHM (50). Since ACPA is elevated even before the onset of RA (51) where Tph cells are dominant, affinity maturation with intensive SHM with the help from Tfh in the GC may be important for the development of ACPA.

The function of Tph cells in autoimmune diseases other than TLS formation and B-cell help is not yet clear. IL-21 produced by Tph cells not only is involved in B-cell help but also may work in dysfunction of vascular endothelial cells (35) and priming of autoreactive CD8^+^ T cells (52). Furthermore, given that a high expression of PD-1 reflects strong activation of T cells by antigens, PD-1^hi^CXCR5^-^CD4^+^ T cells of autoimmune diseases are cells that react strongly to autoantigens and are present in peripheral tissues. Therefore, it is not surprising that Tph cells defined by PD-1^hi^CXCR5^-^ show different characteristics depending on the environment. Indeed, the type I IFN signature is elevated in cTph cells of SLE (27), and STAT3 is elevated in colon Tph cells of Crohn’s disease (53). In IgG4-related diseases, CX3CR1-positive cells express cytotoxic molecules (18). There remains to be determined how these different signatures affect Tph-cell function in the pathogenesis of each disease.

### Tph Responses in Infectious Diseases

Tph cells are increased not only in autoimmune diseases but also in chronic infections such as HIV and chronic hepatitis B (54). As mentioned before, Tph cells are present in lymph nodes of HIV patients and partly share a clonotype with Tfh cells (25). A preprint has shown that Tph is increased in COVID-19 infection and is involved in plasmablast differentiation (55). Even in severe Covid-19 cases, there are upregulated DN2 cells and downregulated SHM in IgG1 (56). These suggests the importance of understanding immune responses orchestrated by Tph cells in infectious diseases and vaccines.

### Tph Responses in Malignant Tumors

In human breast cancer, PD-1^+^ICOS^+^CXCR5^-^CD4^+^ T cells were reported to produce CXCL13 before the proposal of the Tph subset (57). In RA, CXCL13 production is limited to CD4^+^ T cells, whereas in various types of cancers, CD8^+^ cells, in addition to CD4^+^ T cells, also produce CXCL13 (57–59). CXCL13 production by CD8^+^ T cells is also TGF-β dependent like CD4^+^ T cells (60, 61), but less influenced by IL-2 and enhanced in the presence of IL-12 *in vitro* (61). Probably because of Th1/CTL-dominant environment in tumors, intratumoral CD8^+^ T cells are more prone to produce CXCL13. Importantly, the presence of TLS has been associated with a favorable prognosis in patients with various types of cancer (62–64). These suggest that the *in situ* immune response in cancer tissue triggered by TLS formation is involved in cancer immunity. It is interesting to note whether CD4^+^ or CD8^+^ T cells, the source of CXCL13 in tumors, are more involved in TLS formation. Ukita et al. showed that CXCL13^+^CD4^+^ T cells, but not CXCL13^+^CD8^+^ T cells, accumulate in early-stage TLS of ovarian cancer (61). This suggests that PD-1^hi^CXCR5^-^CD4^+^ Tph cells may play an important role in the formation of TLS in tumors. Furthermore, the accumulation of immune cells of multiple species are associated with long-term prognosis (61). These indicate that the progression of acquired immune responses based on TLS is crucial for the establishment of cancer immunity. IL-21 produced by CD4^+^ T cells has been shown to be required for CTL priming in mouse models (65). Similarly human CTL might be primed by IL-21-producing CD4^+^ T cells such as Tph cells in intratumor TLS. CXCL13^+^CD8^+^ T cells express exhaustion markers such as PD-1 (58), but PD-1^+^CD8^+^ T cells also known to exhibit cancer antigen-specific features such as CD103 and CD39 (66, 67). This suggests that CXCL13-producing CD8^+^ T cells could be players crucial for cancer immunity and would be targeted by checkpoint inhibition therapy. Thus, intratumor TLS formation and priming of CTLs by Tph cells play an important role in cancer immunity.

## Closing Remarks

Tph cells were identified from human immunology studies and have been shown to orchestrate immune responses in peripheral tissues. Although the analysis of human immunity has various limitations, advances in research techniques have revealed interesting immune features including the proliferation of B cells with low SHM in peripheral tissues. It is becoming clear that such immune responses are associated with a wide range of diseases. The elucidation of the peripheral immune response orchestrated by Tph cells will lead to a better understanding of the pathogenesis of various diseases and the development of new therapies of these diseases.

## Author Contributions

The author confirms being the sole contributor of this work and has approved it for publication.

## Funding

This work is supported by the Practical Research Project for Allergic Diseases and Immunology from the Japan Agency for Medical Research and Development (AMED: Grant Number JP22ek0410080) and Grants-in-Aid for Scientific Research and Grant-in-Aid for Challenging Research from the Ministry of Education, Culture, Sports, Science and Technology of Japan (Grant Number JP19H03780 and JP20K20610).

## Conflict of Interest

The author declares that the research was conducted in the absence of any commercial or financial relationships that could be construed as a potential conflict of interest.

## Publisher’s Note

All claims expressed in this article are solely those of the authors and do not necessarily represent those of their affiliated organizations, or those of the publisher, the editors and the reviewers. Any product that may be evaluated in this article, or claim that may be made by its manufacturer, is not guaranteed or endorsed by the publisher.
